# What Is the Effect of Inguinal Hernia Operations on Sexual Functions?

**DOI:** 10.7759/cureus.24137

**Published:** 2022-04-14

**Authors:** Hasan Cantay, Mehmet Ezer, Kenan Binnetoglu, Mehmet Uslu, Turgut Anuk, Harun Bayram

**Affiliations:** 1 Department of General Surgery, Kafkas University Faculty of Medicine, Kars, TUR; 2 Department of Urology, Kafkas University Faculty of Medicine, Kars, TUR; 3 Department of General Surgery, Erzurum Training and Research Hospital, Erzurum, TUR; 4 Department of General Surgery, Bingol State Hospital, Bingol, TUR

**Keywords:** lichtenstein technique, surgery, sexual function, hernioplasty, inguinal hernia

## Abstract

Objective: The study aimed to evaluate sexual function before and after inguinal hernia surgery using a standard, internationally approved, patient-administered questionnaire.

Methods: 57 male inguinal hernia cases operated with the Lichtenstein hernioplasty technique were prospectively included in the study. Patients who agreed to participate in the study had the IIEF (International Index of Erectile Function) scoring system form consisting of 15 questions filled in preoperatively, during the first and sixth months after surgery. Patients' age, BMI, comorbidity, employment status, hernia type, hernia size, and single or bilateral hernia were recorded. The relationship between these variables was evaluated by statistical analysis.

Results: A statistically significant difference was found in terms of erectile function, sexual desire, intercourse function, and overall satisfaction, when the preop-postop first month, preop-postop sixth month, and postoperative first month-postop six-month scores were compared (p < 0.05).

Conclusions: Pain and swelling due to an inguinal hernia can negatively affect the sexual functions of the patient, and most of the patients benefit from this after the surgery. Sexual dysfunction may be one of the indications for an inguinal hernia operation.

## Introduction

Sexual life is an important element in human life, as it is in all living things, in terms of both the continuation of the species and the protection of spiritual integrity. According to the WHO, sexual health is defined as “the positive enrichment and strengthening of personality, communication, and love through the physical, spiritual, mental and social treatment of sexual life” [1].

There are many factors that affect sexual function. One of these factors is surgical interventions. Inguinal hernia (IH) operations have an important place among these surgical interventions due to both their anatomical proximity to the genital area and their frequent occurrence. IH is the most common hernia type and is one of the most common operations in General Surgery. IH operation is one of the most performed operations in the United States, with approximately 800,000 cases per year. Lichtenstein hernioplasty, which is a mesh repair method for IH, is still considered the gold standard method [2-4].

In studies on IH, various factors of postoperative quality of life such as pain and recovery have been evaluated recently. However, the number of studies evaluating the quality of sexual life in patients with IH is few. It is also important to evaluate sexual function after an IH operation because the operation is performed in the inguinal region in close proximity to testicular structures and nerves, which are important for sexual function [5,6]. Sexual functions may be affected as a result of tissue hardening due to mesh (due to foreign tissue reaction). However, the presence of IH may adversely affect sexual function due to pain and cosmetic concerns [6-8].

The aim of the study; to evaluate sexual function before and after IH surgery using a standardized, internationally approved, patient-administered questionnaire.

## Materials and methods

The type of the study is a hospital-focused prospective study which was performed by a multidisciplinary team from Kafkas University Faculty of Medicine, Departments of General Surgery and Urology. The study has been approved by the Ethics Committee of Kafkas University Faculty of Medicine (date: 11.03.2021 and no: 80576354-050-99/20). The study was conducted between March 2021 and March 2022. After obtaining permission, 63 male IH cases operated with the Lichtenstein hernioplasty technique in the General Surgery clinic of Kafkas University were prospectively included in the study. In the operation, 6x11 cm polypropylene mesh was used in all patients. Sexually inactive patients (without partners), patients admitted for secondary surgery, and patients younger than 18 years old and over 70 years old were excluded from the study. Six patients who did not come to the postoperative first and sixth-month follow-ups were excluded from the study and the study was completed with 57 patients. IIEF is a self-administered 15-item questionnaire to assess the erectile function of men in the last four weeks (Table 1) [9]. The patients who accepted to participate in the study had the IIEF scoring system form consisting of 15 questions filled in the preoperative period and in the first and sixth month postoperatively. Patients' age, BMI, comorbidity, employment status, hernia type, hernia size, and single or bilateral hernia were recorded.

**Table 1:** International Index of Erectile Function (IIEF) Questionnaire

Main Domains	Questions	Score Range
Erectile function	Q1. Frequency of achieving erections during sexual activity?	0-5
	Q2. Are erections hard enough for penetration after sexual stimulation?	
	Q3. Frequency of penetration?	
	Q4. Frequency of maintaining an erection after penetration?	
	Q5. Ability to maintain an erection until completion of intercourse?	
	Q15. Rate of confidence to achieve and maintain erections?	1-5
Intercourse function	Q6. Frequency of attempts at sexual intercourse?	0-5
	Q7. Intercourse satisfaction for the patient?	
	Q8. Enjoyment of sexual intercourse?	
Orgasmic satisfaction	Q9. Frequency of ejaculation after sexual intercourse or stimulation?	0-5
	Q10. Frequency of orgasm with intercourse or stimulation?	
Sexual desire	Q11. Frequency of sexual desire?	1-5
	Q12. Rate of level of sexual desire?	
Overall satisfaction	Q13. Satisfaction with overall sexual life?	1-5
	Q14. Satisfaction with sexual relationship with the partner?	

"SPSS version 22 for Windows" software package (SPSS Inc., Chicago, IL, USA) was used for statistical analysis. Frequencies, percentage and mean, median were used in the analyses. Preoperatively and postoperatively, IIEF scoring values were compared with the Wilcoxon Signed Ranks Test. P < 0.05 was considered statistically significant.

## Results

The characteristic features of the patients are shown in Table 2. The mean age of the patients was 46.6±13.0, and the median value was 47 (21-68). The mean BMI was 26.16±3.10 kg/m^2^, and the median value was 25.3 (23.0-36.7) kg/m^2^. In terms of age, based on the median value, the number of people aged 47 years and younger was 29 (50.9%), and those over 47 years old were 28 (49.1%). According to the median value in terms of BMI, the number of those who were 25.3 and below was 12 (21.1%) and the number of those who were above 25.3 was 45 (78.9%). While 49 (86.0%) of the patients were working in a job; 8 (14.0%) were not working. Comorbidity was found in 20 patients (35.1%). IHs were classified according to the European Hernia Society (EHS) groin hernia classification. Regarding hernia types, 19 (33.3%) patients had direct, 28 (49.1%) indirect, and 10 (17.5%) combined type hernias. There were unilateral hernias in 49 (86.0%) and bilateral hernias in eight (14.0%) patients. The mean diameter of hernia was 3.02±1.12 cm, and when the mean value was taken as a reference, 33 (57.9%) patients were 3 cm or less and 24 (42.1%) patients were 3 cm or more. No complications other than seroma and hematoma developed in two patients.

**Table 2:** Patient characteristics

Patient characteristics		N	(%)
Age (year)	≤47	29	50.9
	>47	28	49.1
BMI (kg/m^2^)	≤25.3	12	21.1
	>25.3	45	78.9
Comorbidity	Yes	20	35.1
	No	37	64.9
Hernia type	Direct	19	33.3
	İndirect	28	49.1
	Combined	10	17.5
Hernia side	Unilateral	49	86.0
	Bilateral	8	14.0
Hernia size (cm)	≤3	33	57.9
	>3	24	42.1
Working status	Working	49	86.0
	Not working	8	14.0
Total		57	100,0

Changes in IIEF erectile function domain scores before and after IH repair are shown in Table 3. The mean erectile function score was determined as 18.04±4.73, 19.53±3.92, and 21.26±2.86 at preoperatively, postoperatively first month, and sixth months, respectively. When the preop-postoperatively first month, preop-postop sixth month and postoperative first-month-postop sixth-month scores were compared, a statistically significant difference was found (p-values 0.001, 0.001, 0.001, respectively). The mean score in terms of orgasmic satisfaction was 7.18±2.43, 7.26±2.29 and 7.28±2.11, respectively, and no significant difference was found in preoperative and postoperative comparisons (p-values 0.102, 0.201 and 0.748, respectively). The mean preoperative sexual desire score was 6.51±2.16; in the postoperative period, it is 6.93±1.76 at the first month and 7.33±1.52 at the sixth month. When the preoperative and postoperative periods were compared, a significant difference was found (p-values 0.002, 0.001, 0.011, respectively). In terms of Intercourse function scores, there was a postoperative increase, and a significant difference was found in preoperative and postoperative comparisons (p-values 0.018, 0.001, 0.007, respectively). The preoperative scores mean was 7.58±3.86; postoperative first month 8.16±2.95 and postoperative sixth month 8.80±2.34. However, the mean of the overall satisfaction scores were 6.35±2.33 preoperatively, 6.96±1.68, 7.45±1.15 at the postoperative first and sixth months, respectively; increased in the postoperative period and showed a statistically significant difference (p-values 0.005, 0.001, 0.011, respectively). In Table 4, the median and interquartile values of IIEF scores are given.

**Table 3:** Changes in IIEF erectile function domain scores before and after inguinal hernia repair IIEF - International Index of Erectile Function

Evaluated area	(Mean ± Standard Deviation)			P-values		
	Pre-op average score	Post-op 1^st ^month average score	Post-op 6^st ^month average score	Pre-op vs. post-op 1^st ^month	Pre-op vs. post-op 6^st ^month	Post-op 1^st ^month vs. post-op 6^st ^month
Erectile function	18.04±4.73	19.53±3.92	21.26±2.86	0.001	0.001	0.001
Orgasmic satisfaction	7.18±2.43	7.26±2.29	7.28±2.11	0.102	0.201	0.748
Sexual desire	6.51±2.16	6.93±1.76	7.33±1.52	0.002	0.001	0.011
Intercourse function	7.58±3.86	8.16±2.95	8.80±2.34	0.018	0.001	0.007
Overall satisfaction	6.35±2.33	6.96±1.68	7.45±1.15	0.005	0.001	0.011

 

**Table 4:** Median and interquartile values of IIEF scores IIEF - International Index of Erectile Function

	Percentiles	Pre-op score	Post-op 1^st ^month score	Post-op 6^st ^month score
Erectile function	25	14.50	16.50	20.00
	50	20.00	20.00	22.00
	75	22.00	22.00	23.50
Orgasmic satisfaction	25	6.50	6.50	6.50
	50	8.00	8.00	8.00
	75	9.00	9.00	9.00
Sexual desire	25	6.00	6.00	6.00
	50	7.00	7.00	8.00
	75	8.00	8.00	8.50
Intercourse function	25	5.50	6.00	7.00
	50	9.00	9.00	9.00
	75	10.00	10.00	10.00
Overall satisfaction	25	4.00	6.00	7.00
	50	8.00	8.00	8.00
	75	8.00	8.00	8.00

## Discussion

Pain and cosmetic anxiety associated with an IH can negatively affect sexual function. In addition, IH surgeries may impair sexual function by affecting the spermatic cord, testis and scrotum. During IH surgery, vas deferens, testicular arteries and veins may be injured directly, ilioinguinal, iliohypogastric nerves or ramus genitalia branch of genitofemoral nerve may be damaged. There may be reversible complications such as hematoma, seroma and orchitis, and irreversible complications as a result of testicular damage. As a result of these complications that may occur during surgery, sexual functions may deteriorate [8,10-12].

When the literature is reviewed, several studies have evaluated the sexual functions of patients with IIEF scores before and after IH operation. The erectile function score of patients in the literature, in the study by El-Awady et al., was found to be 20.24 preoperatively, 21.54 in the postoperative third month, and 21.44 in the postoperative ninth month [5]. In the study by Giray et al., it was found to be 18.04 preoperatively, 19.53 in the postoperative first month, and 21.26 in the postoperative sixth month [13]. In the study of Tamer et al., it was measured as 21.14 preoperatively and 22.85 during the postoperative third month [14]. In our study, similar to these studies, the erectile function scores were found to be 18.04 preoperatively, 19.53 in the postoperative first month, and 21.26 in the sixth-postoperative month. When the preoperative and postoperative scores were compared significant difference was observed (p: 0.001 when comparing preoperative and postoperative first month, p; 0.001 when comparing preoperative and postoperative sixth month, p: 0.001 comparing postoperative first month and postoperative sixth month).

Considering the sexual desire scores, it was determined that preoperative 6.51, postoperative first month 6.93, postoperative sixth month 7.33. A significant difference was observed when preoperative and postoperative scores were compared (p: 0.002 when preoperative scores and postoperative first-month scores were compared, p: 0.001 when preoperative scores and postoperative sixth-month scores were compared, p: 0.011 when postoperative first-month scores and postoperative sixth-month scores were compared). In studies, a significant difference was found in terms of sexual desire in the postoperative period compared to the preoperative period [5,13,14].

When evaluated in terms of intercourse function scores; In our study, the preoperative score was 7.58, the postoperative first-month score was 8.16, and the postoperative sixth-month score was 8.80. When the preoperative and postoperative scores were compared, it was observed that there was a statistically significant difference. Similar to many studies, a significant increase in sexual satisfaction was observed after the operation [5,13,14].

No statistically significant difference was found in our study in terms of orgasmic satisfaction in preoperative and postoperative comparison (p > 0.05). In the study in which sexual functions were evaluated in patients who underwent Stoppa hernia repair, no significant difference was found in the preoperative and postoperative sixth-month comparison [6]. In the study in which preoperative and postoperative third-month sexual functions were evaluated in patients who underwent hernia repair, no significant difference was found [14]. Similarly, no significant difference was found in the study in which preoperative and postoperative sexual functions were compared at the third and ninth months [5]. Contrary to these studies, a significant increase in orgasmic satisfaction was found in the study of Giray et al. [13].

Considering the general satisfaction scores, it was determined as 6.35 preoperatively, 6.96 in the first month and 7.45 in the sixth month, and a significant increase was detected. When the preoperative and postoperative scores were compared, it was observed that there was a significant difference (p: 0.005 when comparing preoperative scores and post operative first-month scores, p: 0.001 when comparing preoperative scores and post operative sixth-month scores, p: 0.001 when comparing post operative first-month scores and postoperative sixth-month scores). In many studies, general satisfaction increased significantly in the postoperative period [5,13,14].

The inclusion criteria of the study included people at sexually active ages and the exclusion of secondary IH cases from the study had a positive impact on the results. When we look at the literature, when the complications of IH surgery are compared with the benefits of IH surgery, the positive effect of the surgery on sexual functions is higher. We thought that this was due to the reduction of pre-operative pain and the elimination of cosmetic concerns. In addition, in the study conducted by Bulus et al. on 40 patients, they compared testicular arterial blood flow before and after IH surgery and they observed a significant increase in post-operative blood flow [15]. This result shows that the surgery has a positive contribution to sexual functions by increasing testicular blood supply.

Limitations

Apart from organic pathologies, there are many other factors that affect sexual functions, such as psychosocial and sociocultural factors. These factors could not be evaluated during this study. The sample size is not large enough for separate evaluations in subgroups with certain diseases known to be clearly associated with erectile dysfunction, such as diabetes mellitus. For this reason, there is a need for similar studies in larger case series in order to reveal results with a higher level of scientific evidence.

## Conclusions

In conclusion, although the complications that may develop due to inguinal surgery threaten sexual functions, the pain and swelling due to IH itself may negatively affect the sexual functions of the patient. The majority of patients benefit from the reduction of pain and swelling after surgery. This improvement provides an improvement in sexual functions. Sexual dysfunction may be one of the indications for IH operation.
